# The Urokinase Receptor Induces a Mesenchymal Gene Expression Signature in Glioblastoma Cells and Promotes Tumor Cell Survival in Neurospheres

**DOI:** 10.1038/s41598-018-21358-1

**Published:** 2018-02-14

**Authors:** Andrew S. Gilder, Letizia Natali, Danielle M. Van Dyk, Cristina Zalfa, Michael A. Banki, Donald P. Pizzo, Huawei Wang, Richard L. Klemke, Elisabetta Mantuano, Steven L. Gonias

**Affiliations:** 1 Department of Pathology, University of California San Diego, La Jolla, California 92093 USA; 2 Department of Experimental Medicine, Sapienza University of Rome, Rome, Italy

## Abstract

*PLAUR* encodes the urokinase receptor (uPAR), which promotes cell survival, migration, and resistance to targeted cancer therapeutics in glioblastoma cells in culture and in mouse model systems. Herein, we show that patient survival correlates inversely with *PLAUR* mRNA expression in gliomas of all grades, in glioblastomas, and in the subset of glioblastomas that demonstrate the mesenchymal gene expression signature. *PLAUR* clusters with genes that define the more aggressive mesenchymal subtype in transcriptome profiles of glioblastoma tissue and glioblastoma cells in neurospheres, which are enriched for multipotent cells with stem cell-like qualities. When *PLAUR* was over-expressed or silenced in glioblastoma cells, neurosphere growth and expression of mesenchymal subtype biomarkers correlated with uPAR abundance. uPAR also promoted glioblastoma cell survival in neurospheres. Constitutively-active EGF Receptor (EGFRvIII) promoted neurosphere growth; however, unlike uPAR, EGFRvIII did not induce the mesenchymal gene expression signature. Immunohistochemical analysis of human glioblastomas showed that uPAR is typically expressed by a small sub-population of the cancer cells; it is thus reasonable to conclude that this subpopulation of cells is responsible for the effects of *PLAUR* on patient survival. We propose that uPAR-expressing glioblastoma cells demonstrate a mesenchymal gene signature, an increased capacity for cell survival, and stem cell-like properties.

## Introduction

Epithelial-mesenchymal transition (EMT) and mesenchymal-epithelial transition are necessary processes in normal embryogenesis^1^. When a cell acquires a mesenchymal phenotype, it demonstrates increased capacity for cell migration and invasion, resistance to apoptosis, and properties of stem cells^1,2^. EMT has been demonstrated in cancer cells in culture and in pre-clinical animal models of cancer. In these contexts, cancer cells that have undergone EMT demonstrate increased cell migration, invasion, and metastasis^3,4^. Although the significance of EMT in human malignancies has been questioned, EMT has been demonstrated in circulating tumor cells in human blood, indicating that this transformation occurs in human cancers at least under some circumstances^5^. Understanding the molecular pathways that drive EMT in cancer remains an important problem.

The *PLAUR* gene product, uPAR, is a glycosyl-phosphatidylinositol-anchored membrane protein that binds the serine proteinase, urokinase-type plasminogen activator (uPA), and activates a cascade of extracellular proteinases that function in tissue remodeling^6–8^. At the same time, uPAR associates with integrins and receptor tyrosine kinases in the plasma membrane to form a potent multiprotein cell-signaling complex^9–11^. In breast cancer cells, uPAR-activated cell-signaling induces EMT^12,13^, together with many of the changes identified in non-malignant cells that undergo EMT, including increased capacity for cell migration^14,15^, resistance to apoptosis^16–18^, and stem cell-like properties^19^. Although uPA-binding amplifies uPAR-activated cell-signaling and expands the scope of cell-signaling factors activated^9–11,14^, uPAR also signals independently of uPA and promotes cancer metastasis in preclinical animal models when uPA-binding is not possible^20–22^.

Despite recent advances in treatment, grade IV gliomas/glioblastomas still carry a very poor prognosis^23,24^. Genetic, epigenetic, and transcriptome profiling studies have revealed extensive heterogeneity in glioblastomas^25–28^. As a result, attempts have been made to sub-classify these tumors using profiling results. Verhaak *et al.*^28^ analyzed transcriptome profiling data and classified glioblastomas as classical, proneural, neural, or mesenchymal. The mesenchymal subtype of glioblastoma expresses high levels of genes that are biomarkers of EMT and also genes expressed by Schwann cells and microglia. Mesenchymal glioblastomas may have a greater capacity for invasion^29^. Furthermore, the mesenchymal gene expression signature is selectively observed in circulating glioblastoma cells in the blood^30^. One limitation in applying classification schemes in glioblastoma is the diversity in gene expression profiles observed in individual tumor cells in a single cancer^31^. We hypothesized that cells within a glioblastoma that demonstrate a mesenchymal gene expression signature may be the most aggressive.

*PLAUR* has been characterized as a gene expressed selectively by mesenchymal glioblastomas^28^. This is intriguing because, in cell culture and animal model systems, uPAR promotes glioblastoma cell survival, cell migration, and resistance to targeted cancer therapies^32–34^. The role of uPAR in human glioblastoma in patients remains less clearly defined. Herein, we demonstrate that high levels of *PLAUR* mRNA expression correlate inversely with patient survival when Grade II, III, and IV gliomas are considered collectively, when glioblastomas are examined, and when only glioblastomas that express a mesenchymal gene expression signature are examined. In immunohistochemistry (IHC) studies of human glioblastomas, uPAR was robustly expressed by a small sub-population of the cancer cells, suggesting that the effects of *PLAUR* expression on patient survival in glioblastoma may reflect the activity of uPAR in a sub-population of the cancer cells.

To identify pathways by which *PLAUR* gene expression in occasional tumor cells may affect patient survival, we examined glioblastoma cells in neurospheres, which select for multipotent cells with cancer stem cell-like properties^35–37^. We showed that uPAR promotes expression of other genes that serve as biomarkers of the mesenchymal glioblastoma subtype. uPAR also promoted neurosphere growth and inhibited glioblastoma cell apoptosis in neurospheres. These effects were observed even when the glioblastoma cells expressed a constitutively-active variant of the EGF Receptor (EGFRvIII). We propose that *PLAUR* gene expression in glioblastoma adversely affects patient survival by promoting a mesenchymal gene expression profile, by allowing cell survival, and by inducing stem cell-like properties in a small sub-population of glioblastoma cells.

## Results

### *PLAUR* mRNA expression varies with glioma grade and predicts worsened patient survival

**Figure 1:**
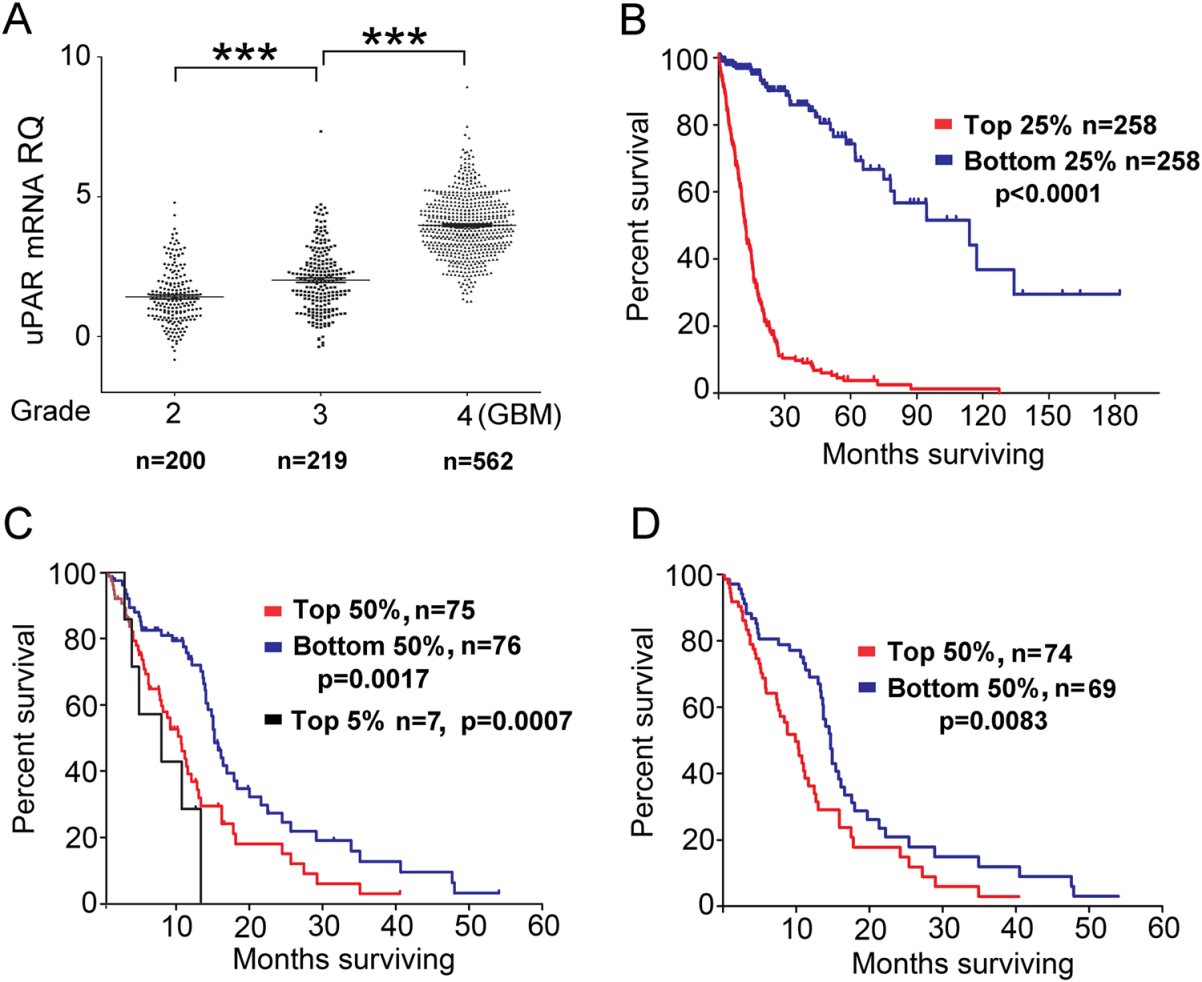
uPAR expression correlates with patient survival in gliomas. (**A**) TCGA data were mined. The relative quantity (RQ) of *PLAUR* mRNA is shown for grade II, III, and IV gliomas (***p < 0.001; one way ANOVA with Bonferroni’s multiple comparison test). (**B**) Kaplan-Meier survival curves were generated from TCGA data, comparing grades II-IV glioma patients collectively. Cases were stratified to compare the 25% in which the tumors demonstrated the highest *PLAUR* mRNA expression levels with the 25% in which the tumors demonstrated the lowest *PLAUR* mRNA expression levels. Statistical significance was determined using the Log-rank test. (**C**) Kaplan-Meier patient survival curves were generated using TCGA RNA-Seq data and comparing glioblastoma cases in which *PLAUR* mRNA expression was above or below the median expression level. Cases in the top 5% for *PLAUR* mRNA expression also were evaluated. Statistical significance was determined by Log-rank test. (**D**) Kaplan-Meier survival curves were generated as described in panel C, but with GCIMP tumors omitted from the analysis.

Yamamoto *et al*.^38^ examined 17 gliomas and first demonstrated that *PLAUR* expression correlates with tumor grade. Salajegheh *et al*.^39^ analyzed 65 diverse primary brain tumors and also noted the association between *PLAUR* expression and tumor grade. In the current study, we mined microarray gene expression data in The Cancer Genome Atlas (TCGA) comparing *PLAUR* mRNA expression in 981 Grade II, III, and IV gliomas^40^. *PLAUR* mRNA expression was significantly increased in Grade III gliomas, compared with Grade II gliomas (p < 0.001), and further increased in Grade IV gliomas/glioblastomas compared with grade III gliomas (p < 0.001) (Fig. 1A). When the cases in this dataset^40^ were stratified according to *PLAUR* mRNA expression and those in the top 25% were compared with those in the bottom 25%, high levels of *PLAUR* mRNA significantly predicted worsened patient survival, as determined by Log-rank test (p < 0.0001) (Fig. 1B).

Next, we analyzed RNA-Seq mRNA expression data in a TCGA dataset of 151 patients with glioblastoma^27^. RNA-Seq measures mRNA quantitatively over an increased dynamic range^41^. Figure 1C shows that, in a cohort consisting exclusively of patients with glioblastoma, patient survival was significantly decreased when *PLAUR* mRNA expression was above the median level compared with cases in which *PLAUR* expression was below the median level (p = 0.0017). Patient survival was further decreased when *PLAUR* mRNA expression was in the top 5% (p = 0.0007). In this dataset, six glioblastomas were characterized as CpG island methylator phenotype (GCIMP) tumors, which are associated with improved patient survival^29,42,43^. In five of six GCIMP tumors, *PLAUR* mRNA expression was below the median value for the entire 151 case dataset. When the GCIMP tumors were omitted, the association between *PLAUR* mRNA expression and decreased patient survival was still observed (p = 0.0083) (Fig. 1D).

### Increased *PLAUR* mRNA expression is associated with decreased patient survival in the mesenchymal subtype of glioblastoma

**Figure 2:**
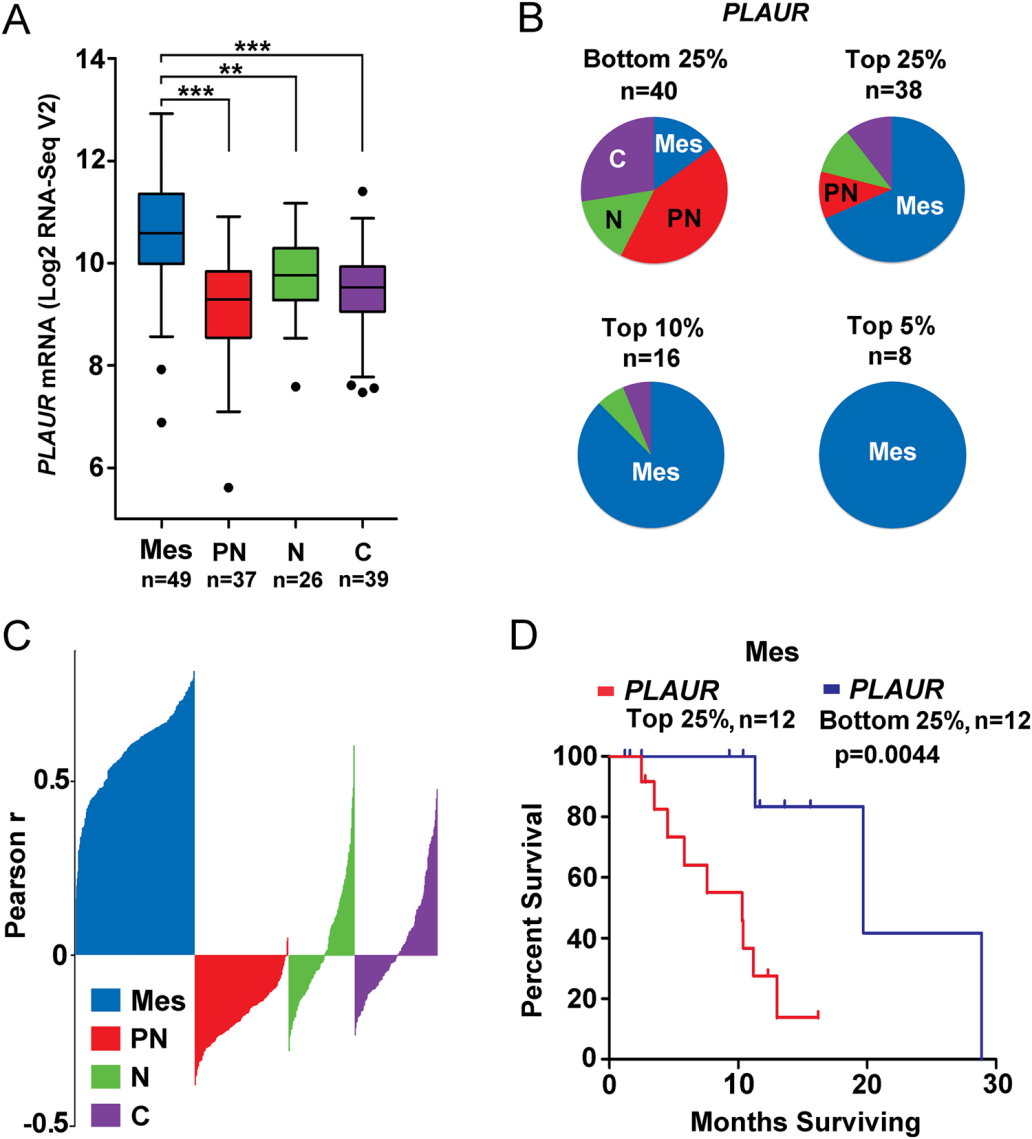
*PLAUR* mRNA expression correlates inversely with patient survival in cases of glioblastoma that demonstrate a mesenchymal gene expression signature. (**A**) Relative *PLAUR* mRNA expression is shown for glioblastomas previously characterized as: mesenchymal (Mes); proneural (PN); neural (N) and classical (**C**) (***p < 0.001; **p < 0.01; one way ANOVA with Tukey’s post hoc test). (**B**) Distribution of glioblastomas into the four subtypes is compared when 151 cases are stratified according to *PLAUR* mRNA expression as follows: 25% of the cases with the lowest level of *PLAUR* mRNA expression; 25% of the cases with the highest level of *PLAUR* mRNA expression; 10% of the cases with the highest level of *PLAUR* mRNA expression and; 5% of the cases with the highest level of *PLAUR* mRNA expression. (**C**) Silhouette plot showing correlations between *PLAUR* expression and 685 genes that serve as biomarkers of the four glioblastoma subtypes. The plot is a condensed series of bar graphs corresponding with ascending Pearson r-values for each subtype. Regions above the x-axis demonstrate a positive correlation. Regions below indicate a negative correlation. (**D**) Analysis of patient survival as a function of *PLAUR* mRNA expression in glioblastoma cases classified as mesenchymal (Mes). Kaplan-Meier patient survival curves were generated comparing 25% of the cases in which the tumors demonstrated the highest *PLAUR* mRNA expression levels with 25% of the cases in which the tumors demonstrated the lowest *PLAUR* mRNA expression levels. Statistical significance was determined by Log-rank test.

Analysis of RNA-Seq data^27^ demonstrated that *PLAUR* mRNA expression is significantly increased in the mesenchymal subtype of glioblastoma, compared with the proneural (p < 0.0001), neural (p < 0.001), and classical (p < 0.001) subtypes of glioblastoma (Fig. 2A,B). A silhouette plot shows the strong positive correlation between *PLAUR* mRNA expression and expression of other mesenchymal signature genes (Fig. 2C). Positive and negative correlations (above and below the baseline) were observed when *PLAUR* mRNA expression was compared with genes that define the neural and classical subtypes. A profoundly negative correlation was observed when *PLAUR* was compared with genes that define the proneural subtype.

To assure that *PLAUR* was not influencing patient survival in glioblastoma because it serves as a surrogate biomarker of other co-regulated genes in the mesenchymal gene signature, we analyzed patient survival selectively in mesenchymal glioblastomas^27^. Figure 2D shows that when patients with the mesenchymal subtype of glioblastoma were stratified according to *PLAUR* mRNA expression and those in the top 25% were compared with those in the bottom 25%, high levels of *PLAUR* mRNA significantly predicted worsened patient survival, as determined by Log-rank test (p = 0.004).

### uPAR protein is expressed by a sub-population of cancer cells in human glioblastomas

**Figure 3:**
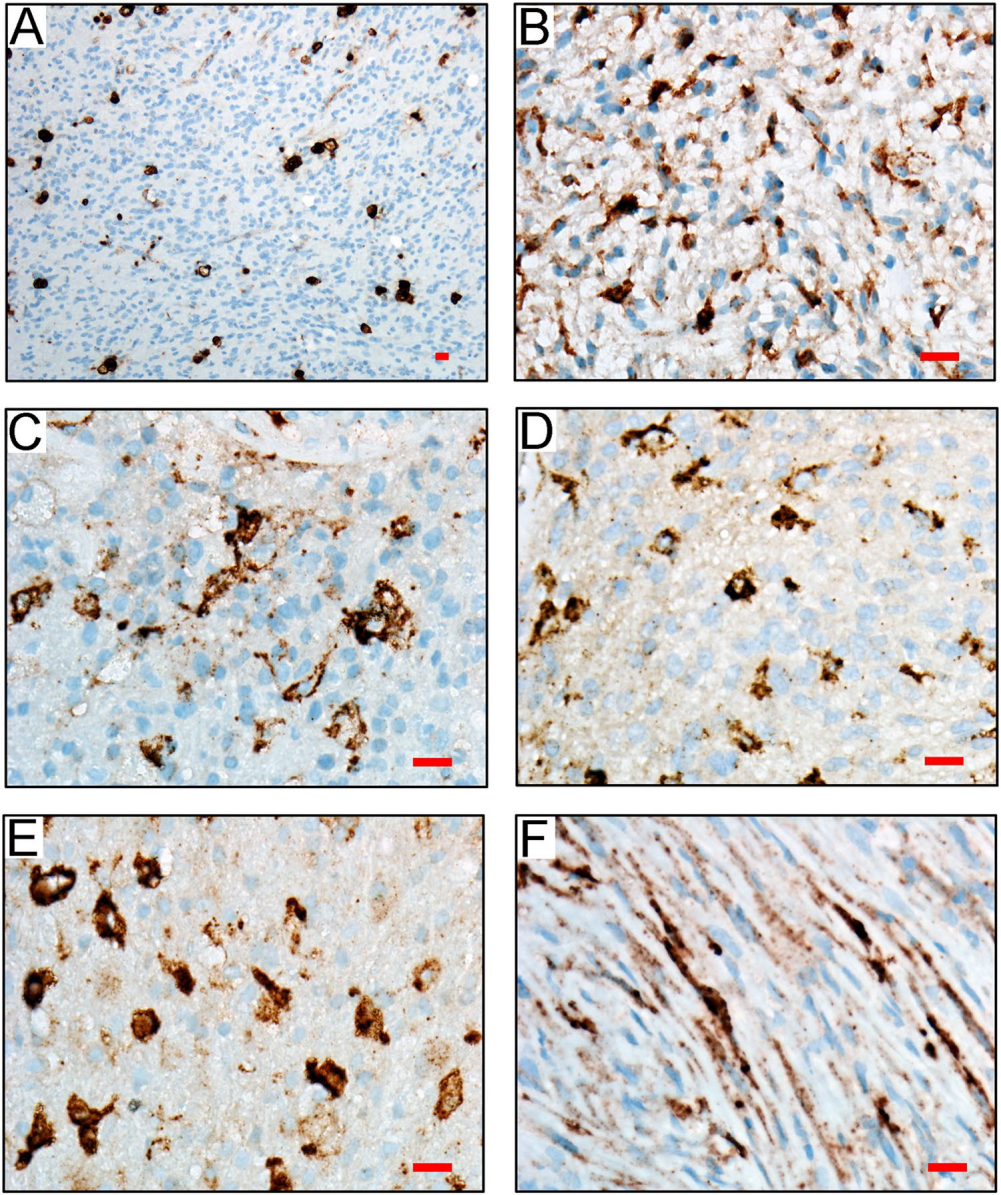
In human glioblastomas, uPAR protein is expressed by a sub-population of the tumor cells. (**A**–**F**) Representative photomicrographs showing uPAR IHC with hematoxylin counterstain in randomly selected glioblastomas (scale bar, 50 µm). The images shown in panels A and B are from the same tumor. Panel B is shown to demonstrate a region of this tumor in which the density of uPAR-immunopositive cells is much higher than in other regions. Panel F shows a region of a glioblastoma in which the cells have adopted a spindle cell morphology. In this area, we also observed a higher than average number of uPAR-immunopositive cells.

Given the correlation between *PLAUR* mRNA expression and patient survival in glioblastoma, we undertook studies to examine uPAR protein expression in human glioblastomas by immunohistochemistry (IHC). We examined 14 tumors, without patient identifiers, surgically resected at the University of California San Diego Hospital and diagnosed by clinical neuropathologists. uPAR was detected in at least some cancer cells in each specimen; however, the frequency of uPAR-immunopositive cells was variable from tumor to tumor and within different regions of the same tumor. Most frequently, uPAR immunopositive tumor cells were embedded amongst numerous uPAR-immunonegative cells, as determined by hematoxylin counterstaining (Fig. 3A). The high power field shown in Fig. 3B is from the same tumor imaged in Fig. 3A; the area imaged in Fig. 3B shows a substantially increased density of uPAR-immunopositive cells, compared with other regions of the same tumor. Images of four other representative tumors are shown in Fig. 3C–F. The image shown in Fig. 3F shows an area of a glioblastoma in which the tumor cells adopted spindle-cell morphology and also were robustly uPAR-immunopositive. We previously demonstrated that uPAR immunostaining is fairly uniform amongst human glioblastoma cells that are propagated in xenografts^32^. The studies presented here show that in resected human glioblastomas, prior to xenografting, uPAR immunostaining is quite heterogeneous. Furthermore, these results suggest that the correlation between *PLAUR* mRNA expression and patient survival in glioblastoma may reflect the activity of uPAR protein in a small sub-population of the cancer cells.

### *PLAUR e*xpression is increased in glioblastoma cells that express the mesenchymal gene signature in neurosphere culture

To determine how *PLAUR* expression in a small sub-population of glioblastoma cells may influence tumor aggressiveness, we studied glioblastoma cells in neurosphere culture. This culturing method selects for glioblastoma cells with tumor-initiating or cancer stem cell-like properties; glioblastoma cells in neurospheres have an increased capacity for self-renewal and the ability to recapitulate the heterogeneity in the original tumor when transplanted into mice^35–37,44,45^. Neurosphere culture eliminates contamination by non-tumor cells, which contributes to the results obtained when gene expression is examined in intact tumor samples.

**Figure 4:**
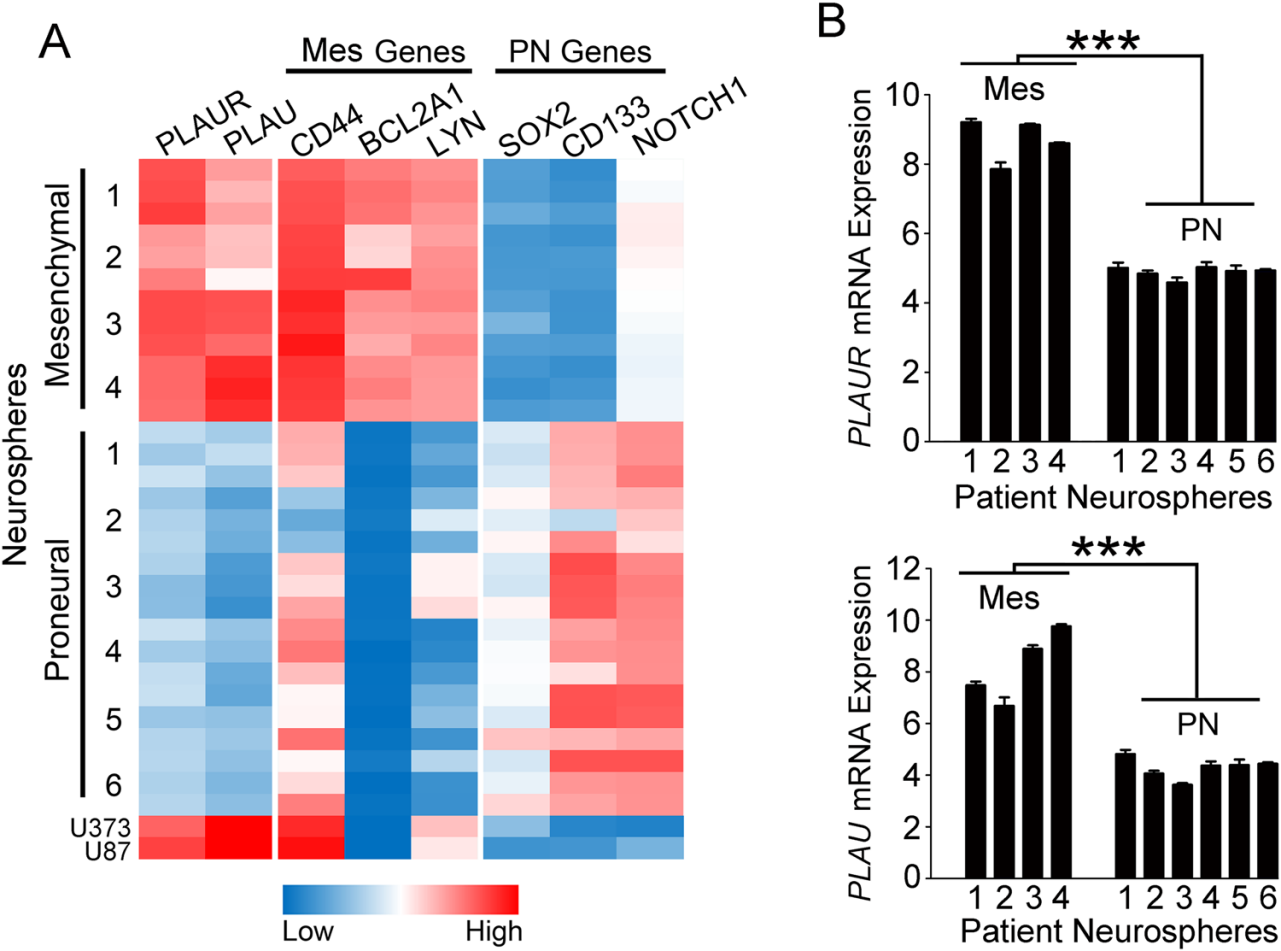
Database mining reveals that *PLAUR* mRNA is expressed selectively by glioblastoma cells that demonstrate a mesenchymal gene expression signature when propagated in neurospheres. (**A**) The dataset presented by Mao *et al*.^46^ was mined to generate a heatmap showing microarray gene expression data for tumor cells in neurospheres, established using ten separate cases of glioblastoma. Expression is shown for *PLAU*, *PLAUR* and genes known to serve as biomarkers of the proneural (PN) and mesenchymal (Mes) subtypes of stem-like cells in neurospheres. (**B**) Absolute mRNA expression is shown for *PLAUR* and *PLAU* in extracts of cells from neurospheres with mesenchymal and proneural gene expression signatures (false discovery rate method, ***p < 0.001).

Mao *et al*.^46^ profiled the transciptomes of ten neurosphere cultures established with cells isolated from different surgically-resected human glioblastomas and characterized the cells in these neurospheres as mesenchymal or proneural. Cells in neurospheres with mesenchymal gene expression signatures were more aggressive and radiation-resistant. Prominent biomarkers of mesenchymal glioblastoma cells in neurosphere culture included *CD44*, *BCL2A1*, and *LYN*^46–48^. Biomarkers of the proneural subtype included *SOX2*, *CD133*, and *NOTCH1*^46–48^. We mined the dataset collected by Mao *et al*.^46^, and demonstrated that *PLAUR* mRNA expression clearly sorts with genes that define the mesenchymal subtype (Fig. 4A). *PLAU*, which encodes the ligand for uPAR, urokinase-type plasminogen activator (uPA), also sorted with the mesenchymal gene signature. Figure 4B shows that expression of *PLAU* and *PLAUR* in glioblastoma cells in mesenchymal neurospheres was significantly increased compared with cells in proneural neurospheres, as determined by the false discovery rate method (p < 0.001).

**Figure 5:**
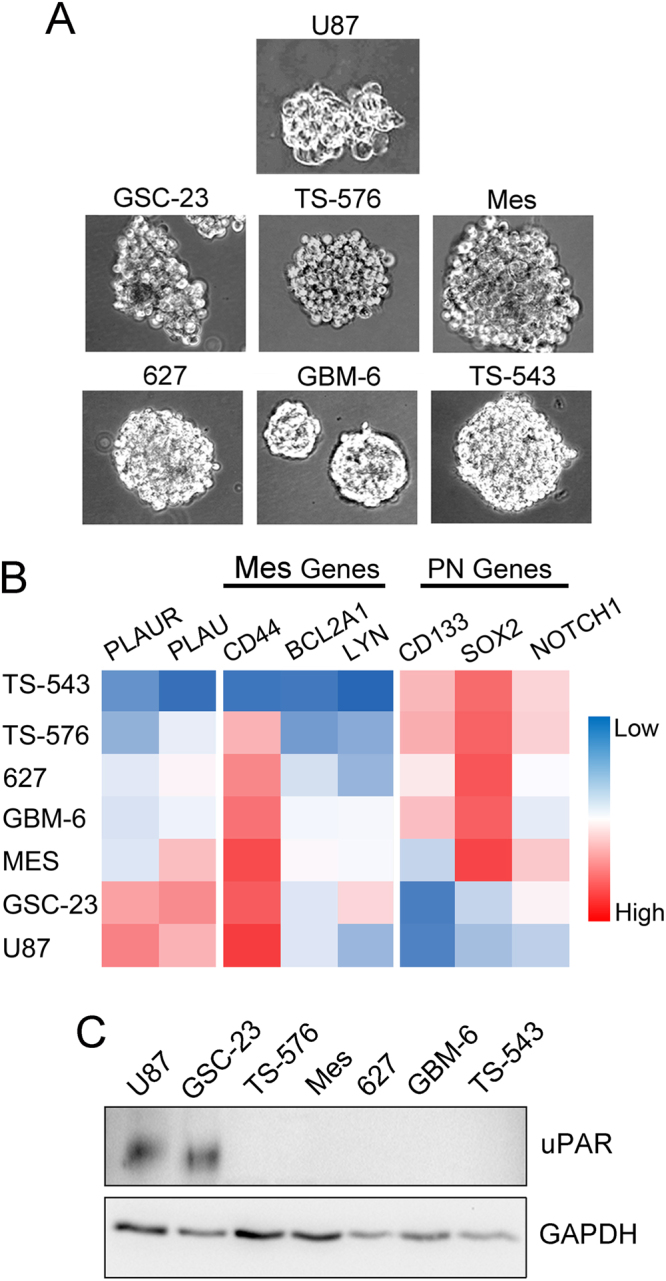
Experimental confirmation of *PLAUR* expression in mesenchymal glioblastoma cells in neurospheres. (**A**) Representative phase-contrast images of neurospheres formed with each of the cell types under investigation. (**B**) Heatmap comparing the relative expression of *PLAUR*, *PLAU*, and biomarkers of the mesenchymal (Mes) and proneural (PN) subtypes of cells in neurospheres, as determined by RT-qPCR. (**C**) Protein extracts were prepared from cells in neurospheres. Immunoblot analysis was performed to detect uPAR. The blot was re-probed for GAPDH as a loading control.

Next, we established neurosphere cultures with GSC-23, TS-576, Mes, 627, GBM-6, and TS-543 glioblastoma cells. All six cell lines had been passaged exclusively under serum-free neurosphere conditions after isolation from human tumors. U87 cells also were established in neurosphere culture. U87 cells in neurosphere culture demonstrate increased multi-potency and capacity for self-renewal and thus, represent a model for glioblastoma cells with stem cell-like properties^49^. Representative images of neurospheres formed by the seven different cell types are shown in Fig. 5A.

RNA was isolated from cells in neurospheres. RT-qPCR was performed to profile expression of genes identified as biomarkers of the mesenchymal and proneural subtypes. Figure 5B shows that GSC-23 and U87 cells demonstrated gene expression profiles most consistent with the mesenchymal subtype. TS-543 cells demonstrated an expression profile most consistent with the proneural subtype. The other cells showed mixed gene expression profiles. GSC-23 cells and U87 cells demonstrated the highest levels of mRNA for *PLAUR* and *PLAU*. We confirmed the *PLAUR* expression results, at the protein level, by immunoblot analysis. uPAR was detected in protein extracts from GSC-23 and U87 cells but not in comparably isolated protein extracts from the other cells (Fig. 5C, Supplemental Fig. 1).

### uPAR regulates expression of genes that define the mesenchymal subtype of glioblastoma

**Figure 6:**
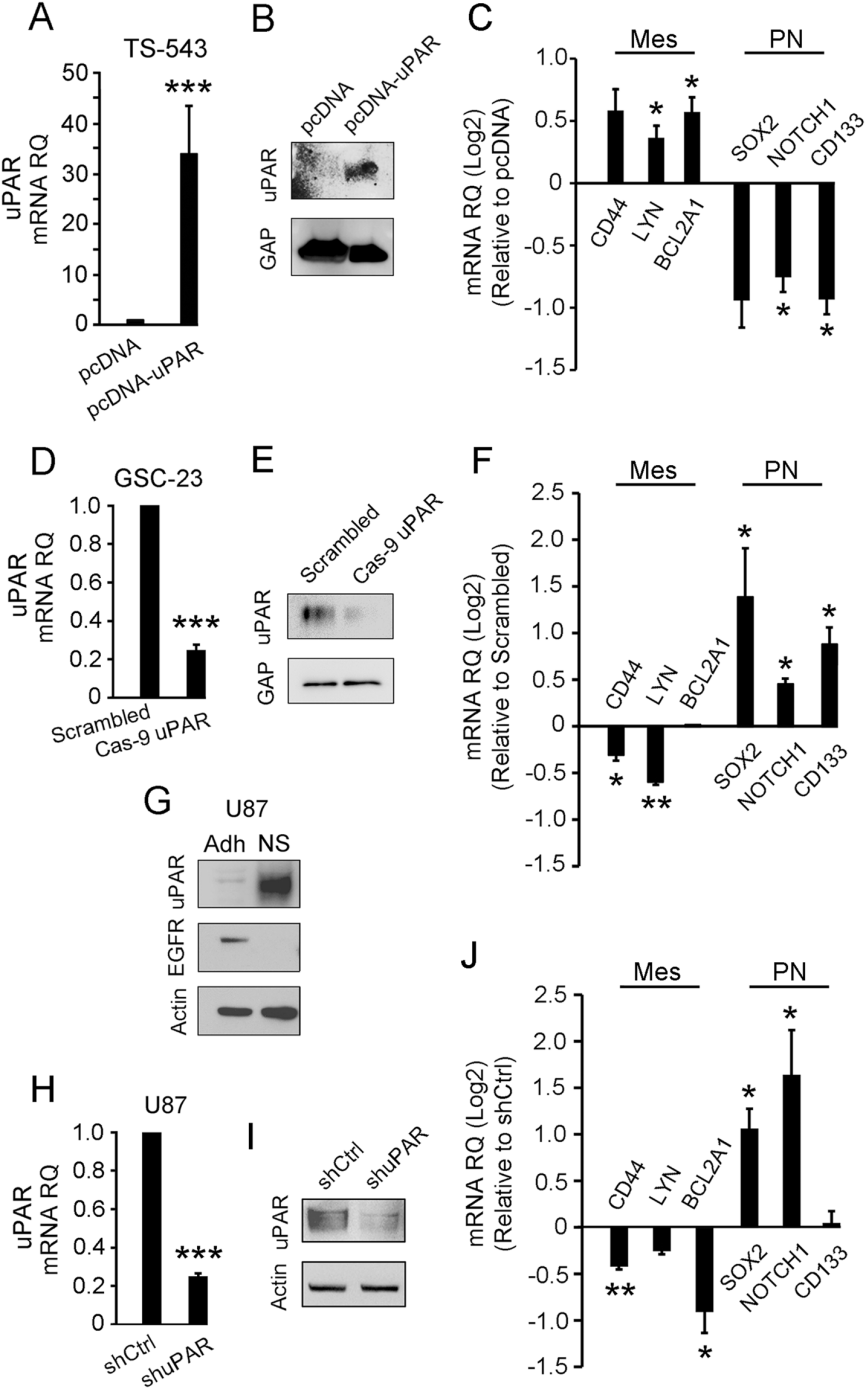
uPAR drives expression of the mesenchymal gene signature in glioblastoma cells in neurospheres. (**A**) RT-qPCR was performed to determine relative *PLAUR* mRNA expression in TS-543 cells in neurospheres transfected with pcDNA-uPAR to over-express *PLAUR* or with empty vector (mean ± S.E., n = 3; student’s t-test, ***p < 0.001). (**B**) Immunoblot analysis was performed to detect uPAR in TS-543 cells in neurospheres transfected with pcDNA-uPAR or empty vector. GAPDH (GAP) was assessed as a loading control. (**C**) RT-qPCR was performed to assess relative mRNA expression of *CD44*, *LYN*, *BCL2A1*, *SOX2*, *NOTCH1* and *CD133* following transfection with pcDNA-uPAR, compared with empty vector (mean ± S.E., n = 3; student’s t-test, *p < 0.05). (**D**) RT-qPCR was performed to determine relative *PLAUR* expression in GSC-23 cells in neurospheres transfected with a *PLAUR-*targeting cas-9 vector or a scrambled vector (scrambled) (mean ± S.E., n = 3; student’s t-test ***p < 0.001). (**E**) Immunoblot analysis was performed to detect uPAR in GSC-23 cells in neurospheres transfected with the *PLAUR-*targeting cas-9 vector or the scrambled vector. GAPDH (GAP) was assessed as a loading control. (**F**) RT-qPCR was performed to determine relative mRNA expression of *CD44*, *LYN*, *BCL2A1*, *SOX2*, *NOTCH1* and *CD133* in GSC-23 cells in neurospheres transfected with the *PLAUR-*targeting cas-9 vector versus the scrambled vector (mean ± S.E., n = 3; student’s t-test **p < 0.01; *p < 0.05). (**G**) Immunoblot analysis was performed to compare uPAR and EGFR protein expression in U87 cells cultured in monolayers in serum-supplemented medium (Adh) or in neurospheres in defined serum-free medium (NS). Actin was used as a loading control. (**H**) RT-qPCR was performed to determine relative *PLAUR* mRNA expression in U87 cells cultured in neurospheres. Cells in which uPAR gene expression was silenced with uPAR-targeting shRNA (shuPAR) and control cells (shCtrl) are compared (mean ± S.E., n = 3; student’s t-test ***p < 0.001). (**I**) Immunoblot analysis showing uPAR protein in U87 cells cultured in neurospheres. Cells transfected with shuPAR or shCtrl are compared. The blot was re-probed for actin as a loading control. (**J**) RT-qPCR was performed to determine relative mRNA expression for *CD44*, *LYN*, *BCL2A1*, *SOX2*, *NOTCH1* and *CD133* in U87 neurospheres transfected with shuPAR versus shCtrl (mean ± S.E., n = 3; student’s t-test **p < 0.01; *p < 0.05).

Because uPAR is a cell-signaling receptor^9–11^, we tested whether uPAR regulates expression of other genes that define the mesenchymal subtype in glioblastoma cells in neurospheres. First, we over-expressed uPAR in TS-543 cells by transfecting cells in neurospheres. Figure 6A shows that uPAR mRNA was increased more than 30-fold (p < 0.001). uPAR protein also was increased, as determined by immunoblot analysis (Fig. 6B). uPAR over-expression significantly increased expression of mRNAs that are biomarkers of the mesenchymal subtype and decreased expression of mRNAs that are biomarkers of the proneural subtype (Fig. 6C).

Next, we blocked uPAR expression in GSC-23 cells using a *PLAUR-*targeting cas-9 vector. Control cells were transfected with a scrambled cas-9 vector. By RT-qPCR, the *PLAUR-*targeting cas-9 vector decreased *PLAUR* mRNA expression in GSC-23 cells in neurospheres by 75 ± 10% (Fig. 6D). uPAR protein expression also was decreased, as determined by immunoblot analysis (Fig. 6E). GSC-23 cells in neurospheres, in which uPAR expression was blocked using the cas-9 vector, demonstrated significantly decreased expression of biomarkers of the mesenchymal subtype and significantly increased expression of biomarkers of the proneural subtype (Fig. 6F).

Finally, we tested the effects of uPAR on gene expression in U87 cells in neurospheres. Figure 6G shows that culturing U87 cells in neurospheres substantially increased uPAR protein expression compared with that detected in U87 cells in monolayer culture. Silencing *PLAUR* gene expression with shRNA decreased *PLAUR* mRNA in U87 cells in neurospheres by 75 ± 6% (Fig. 6H). uPAR protein also was decreased (Fig. 6I). As shown in Fig. 6J, *PLAUR* gene-silencing significantly decreased expression of biomarkers that define the mesenchymal subtype and significantly increased expression of biomarkers that define the proneural subtype in U87 cells in neurospheres. Thus, in several distinct model systems, increased *PLAUR* expression favors the mesenchymal subtype of glioblastoma cell in neurospheres whereas decreased *PLAUR* expression favors the proneural subtype.

### EGFRvIII promotes growth of neurospheres but does not negate the role of uPAR in determining glioblastoma subtype

**Figure 7:**
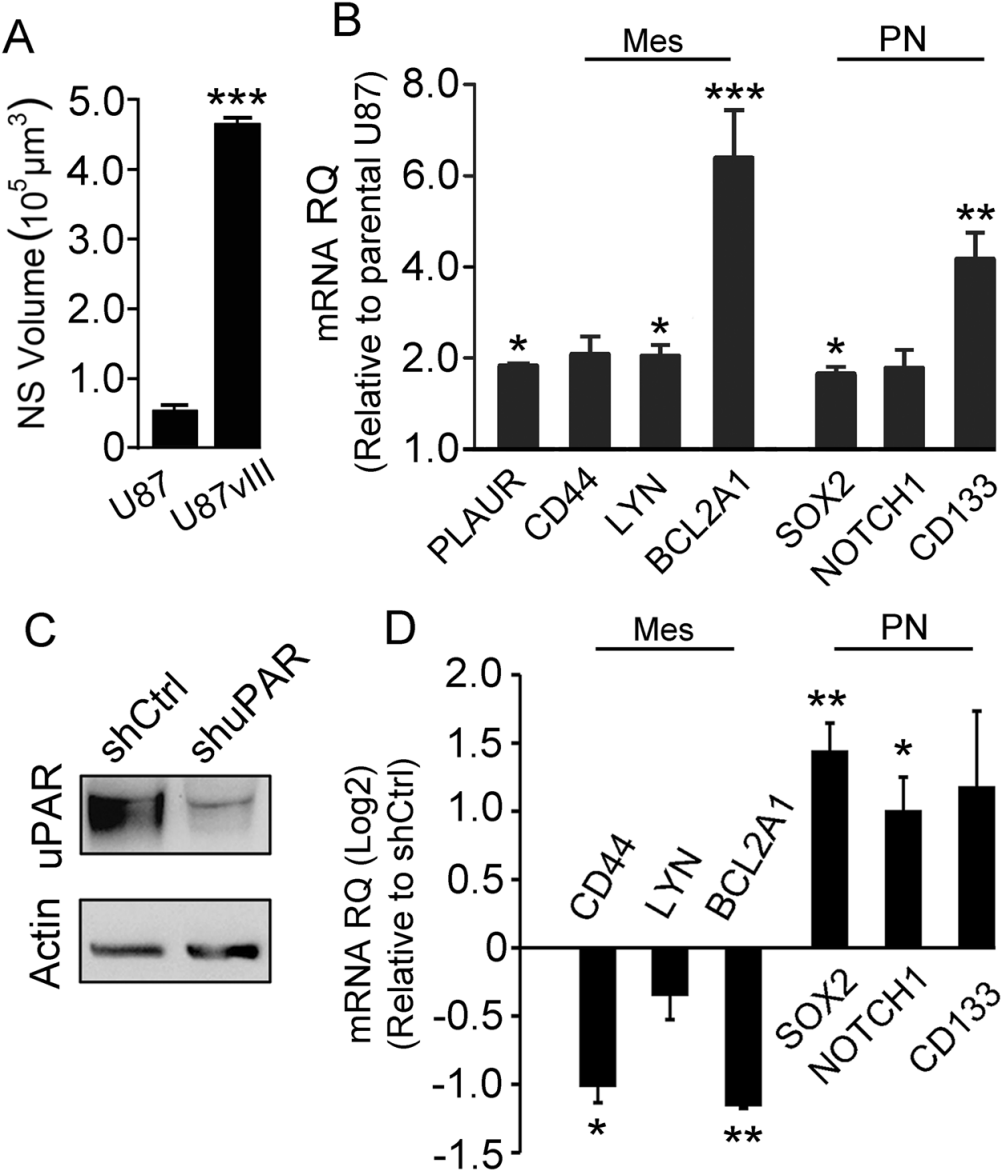
EGFRvIII promotes neurosphere growth but does not block the activity of uPAR in determining the glioblastoma cell subtype in neurosphere cultures. (**A**) U87 and U87vIII cells were cultured under serum-free neurosphere forming conditions for 6 days. The mean neurosphere volume was determined (mean ± S.E.; n = 30; student’s t-test ***p < 0.001). (**B**) RT-qPCR was performed to determine relative expression of *CD44*, *LYN*, *BCL2A1*, *SOX2*, *NOTCH1* and *CD133* in U87vIII cells in neurospheres versus parental U87 cells in neurospheres (mean ± S.E., n = 3; student’s t-test ***p < 0.001; **p < 0.01; *p < 0.05). (**C**) Immunoblot analysis showing uPAR protein in U87vIII cells following gene silencing with shRNA (shuPAR) versus cells that express control shRNA (shCtrl). **(D**) RT-qPCR was performed to determine relative expression of *CD44*, *LYN*, *BCL2A1*, *SOX2*, *NOTCH1* and *CD133* in U87vIII cells that express shuPAR versus shCtrl in neurospheres (mean ± S.E., n = 3; student’s t-test **p < 0.01; *p < 0.05).

The gene that encodes the EGF Receptor (*EGFR*) is over-expressed in up to 50% of all glioblastomas and in many of these tumors, *EGFR* is mutated to generate a constitutively active derivative called EGFRvIII^50,51^. U87 cells that express EGFRvIII are previously characterized and we previously demonstrated that these cells form neurospheres^52,53^. To determine whether activated EGFR signaling affects glioblastoma neurosphere formation, we prepared neurospheres with U87 and U87vIII cells under equivalent conditions. Figure 7A shows that neurospheres formed by U87vIII cells grew significantly larger than neurospheres formed by U87 cells (p < 0.001). EGFRvIII modestly increased *PLAUR* expression by U87 cells in neurospheres and also increased expression of biomarkers of both the mesenchymal and proneural subtypes (Fig. 7B). However, EGFRvIII failed to change the gene expression profile in a manner that would suggest a change in glioblastoma cell subtype.

Next, we silenced *PLAUR* gene expression in U87vIII cells. Figure 7C confirms that *PLAUR* gene-silencing decreased uPAR protein in U87vIII cells in neurospheres. *PLAUR* gene-silencing also decreased expression of biomarkers of the mesenchymal subtype and increased expression of biomarkers of the proneural subtype (Fig. 7D). Thus, uPAR functions as a major regulator of glioblastoma subtype in neurosphere culture even when EGFR signaling is constitutively activated.

The mRNA-targeting sequence in our *PLAUR*-specific shRNA corresponds to nucleotides 397–419 in exon 4 of the *PLAUR* gene. As a control for off-target effects, we obtained siRNA with the equivalent *PLAUR*-targeting sequence (siRNA1) and a second siRNA that targets a distinct sequence in exon 3 of the *PLAUR* gene (siRNA2). The two *PLAUR*-specific siRNAs and non-targeting control (NTC) siRNA were transfected into U87vIII cells in neurospheres. By RT-qPCR, *PLAUR* mRNA expression was decreased by 24 ± 9% by siRNA1 (n = 3) and by 38% ± 3% for siRNA2. Although these levels of gene-silencing were substantially lower than those observed when we transfected monolayer cultures, both *PLAUR*-specific siRNAs generated similar changes in gene expression by U87vIII cells in neurospheres. Biomarkers of the mesenchymal subtype were significantly decreased and biomarkers of the proneural subtype were increased (Supplemental Fig. 2). These results, obtained with different siRNAs, support the conclusion that our results with *PLAUR-*targeting shRNA reflect effects on uPAR expression and not off-target effects.

### uPAR promotes glioblastoma cell survival in neurospheres

**Figure 8:**
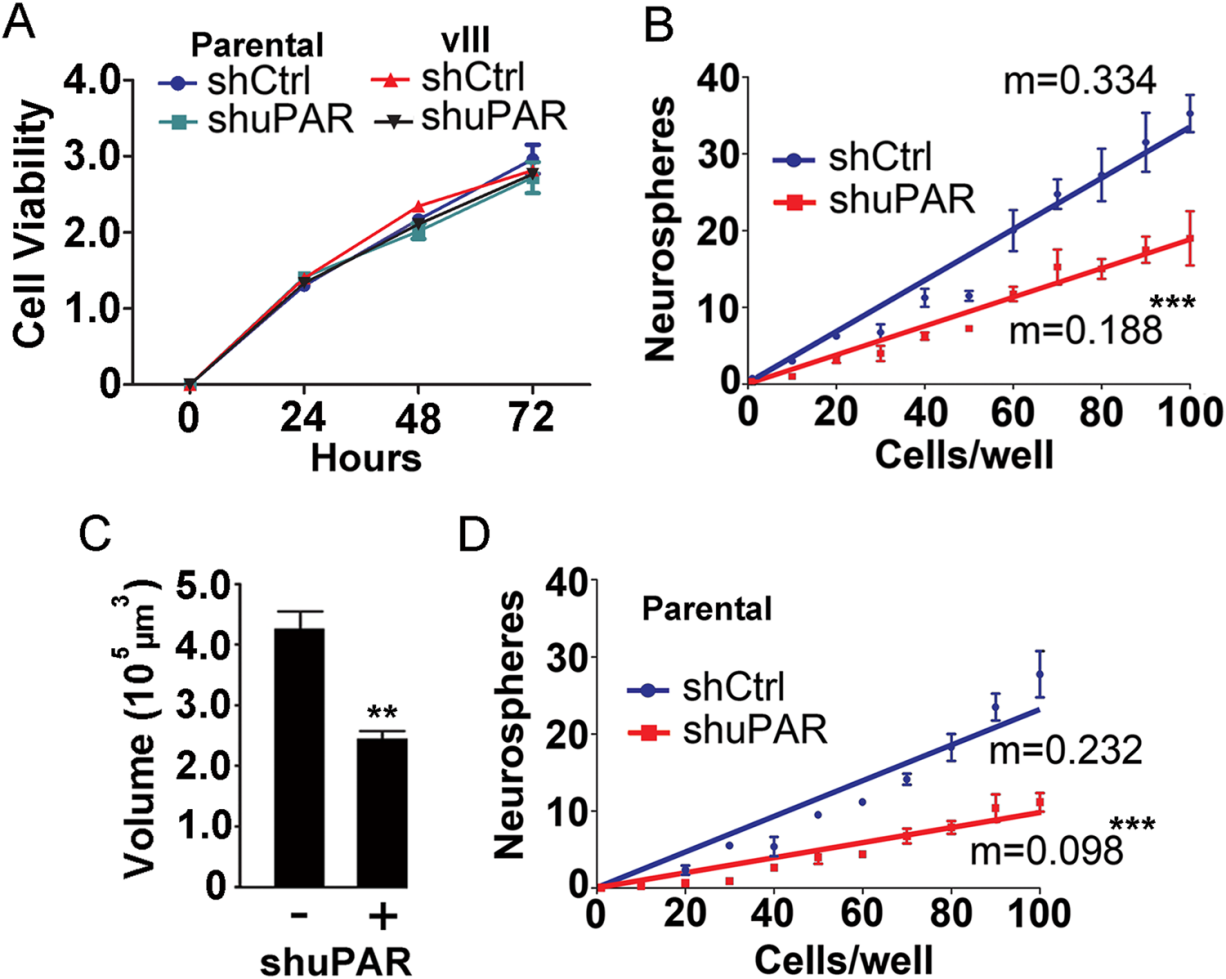
Neurosphere formation and/or growth are regulated by uPAR. (**A**) U87 cells and U87vIII cells that express control shRNA (shCtrl) or uPAR-targeting shRNA (shuPAR) were maintained in monolayer culture in 10% serum-containing medium for up to 72 h. The number of viable cells was quantified by WST-1 assay (mean ± S.E.; n = 3). (**B**) U87vIII cells (10–100/well) were seeded into wells under neurosphere culturing conditions. The number of neurospheres that achieved a minimum average diameter of 60 µm within 14 days is shown (mean ± SEM, n = 4). The slopes from a linear regression were calculated from each individual experiment and determined to be significantly different by student’s t-test (***p < 0.001). (**C**) U87vIII cells that express control shRNA (shuPAR -) or *PLAUR*-targeting shRNA (shuPAR+) were cultured under serum-free neurosphere forming conditions for 6 days. Volume analysis was performed (mean ± SEM; n = 30; student’s t-test **p < 0.01). (**D**) U87 cells that express shCtrl or shuPAR were seeded into neurosphere culture medium. The number of neurospheres was determined as described in panel B (***p < 0.001).

The rapid rate of growth of neurospheres formed by u87vIII cells provided an opportunity to examine the effects of uPAR on the physiology of glioblastoma cells in neurospheres at the cellular and biochemical levels. To begin, U87vIII cells in which *PLAUR* was silenced with shRNA and cells transfected with empty vector were seeded in monolayer culture in serum-supplemented medium (10,000 cells/well). The increase in the number of viable cells was measured as a function of time by WST-1 assay. Under these conditions, *PLAUR* gene-silencing had no effect on cell growth at 24, 48, or 72 h (Fig. 8A).

Next, 10–100 U87vIII cells in which *PLAUR* was silenced or control cells were added to each well of a 96-well plate and cultured in serum-free neurosphere culture medium. After 14 days, the number of neurospheres that formed and attained a minimum diameter of 60 μm was counted. Figure 8B shows that *PLAUR* gene-silencing significantly reduced the neurosphere count over the inoculation range compared with control cells, as determined by comparing the slopes calculated by linear regression (student’s t-test; p < 0.001). Neurospheres formed by *PLAUR* gene-silenced U87vIII cells also demonstrated a significantly decreased mean volume (Fig. 8C). A decrease in the frequency of neurosphere formation in a limiting dilution assay is frequently interpreted as indicating a decrease in the percentage of tumor-initiating cells or cells with stem cell-like properties^35,36,44^; however, the decrease in neurosphere formation also may reflect the effects of uPAR gene-silencing on cell proliferation and/or survival so that neurospheres fail to achieve the 60 µm diameter threshold. Figure 8D shows that *PLAUR* gene-silencing in parental U87 cells (no EGFRvIII expression) significantly decreased the frequency of neurosphere formation (student’s t-test; p < 0.001), mimicking the result observed in U87vIII cells.

**Figure 9:**
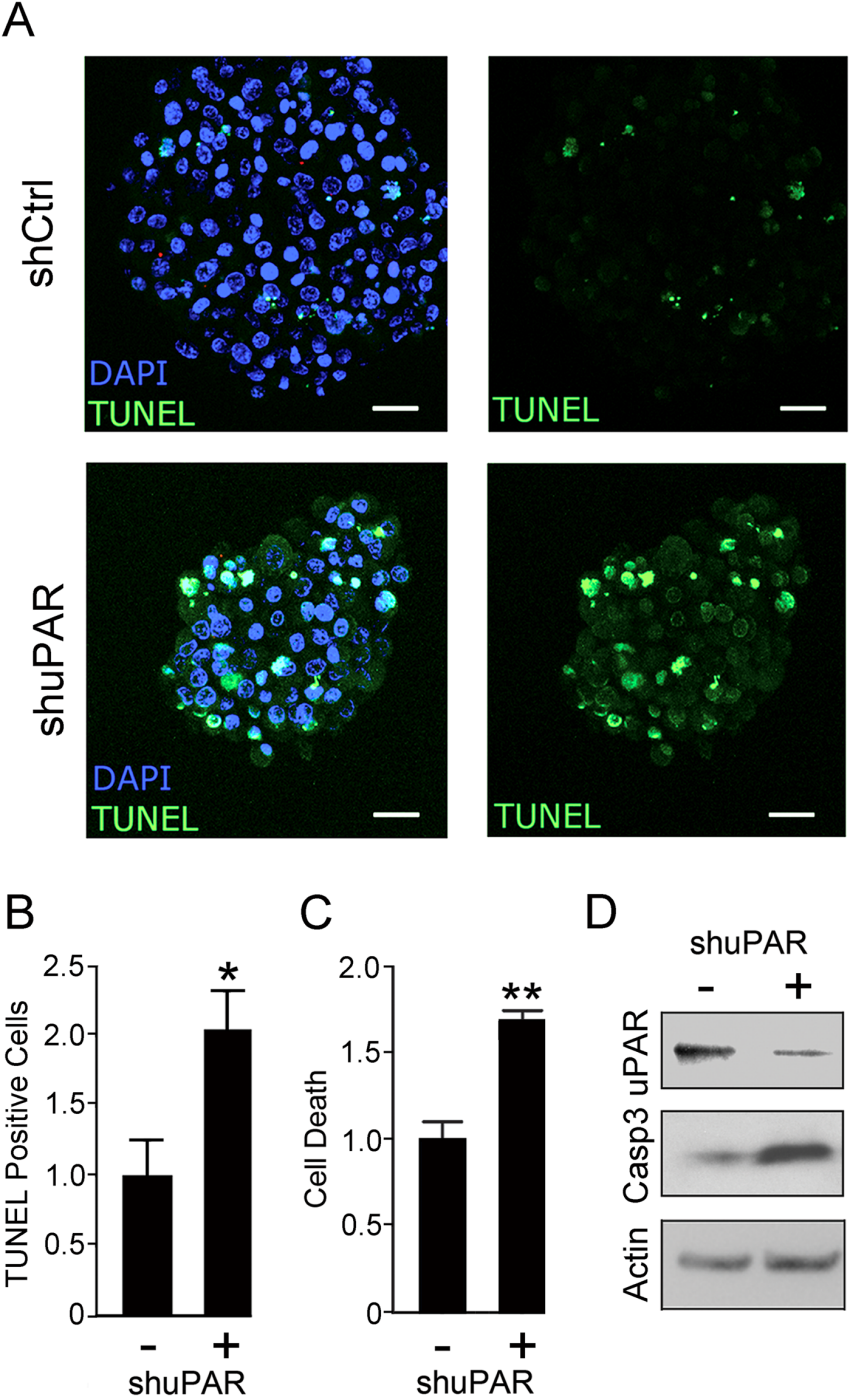
uPAR promotes survival of U87vIII glioblastoma cells in neurospheres. (**A**) Representative confocal fluorescent micrographs of U87vIII neurospheres stably expressing control shRNA (shCtrl) or *PLAUR*-targeting shRNA (shuPAR). The neurospheres were stained for TUNEL (green) and DAPI (blue). (**B**) Quantification of TUNEL-positive cells in control cultures (−) and in cultures of cells that express *PLAUR*-targeting shRNA (+). The ratio of TUNEL-positive cells to the total number of cells in the neurosphere was computed and is presented relative to that present in control cells that express shCtrl (mean ± SEM; n = 5; student’s t-test *p < 0.05). (**C**) U87vIII cells in neurospheres were extracted and studied using the Cell Death ELISA (mean ± SEM; n = 3; student’s t-test **p < 0.01, *p < 0.05). (**D**) U87vIII cells that express shuPAR or shCtrl in neurospheres were extracted. Immunoblot analysis was performed to detect uPAR, cleaved caspase-3 (Casp3), and actin to control for load.

To determine whether *PLAUR* gene-silencing affects cell survival in neurospheres, first we performed TUNEL staining on intact neurospheres. U87vIII cells in which *PLAUR* was silenced with shRNA and control cells were compared. Figure 9A shows that TUNEL-positive cells were readily detected in neurospheres when *PLAUR* was silenced. The number of cells that were TUNEL-positive was determined as a percentage of the DAPI-stained nuclei. As shown in Fig. 9B, the number of TUNEL-positive cells was significantly increased in neurospheres formed by *PLAUR* gene-silenced cells (p < 0.05).

Next we isolated cells from neurospheres and quantified intracytoplasmic oligonucleosomes, which accumulate in apoptotic cells, by ELISA. Figure 9C shows that *PLAUR* gene-silencing significantly increased intracytoplasmic oligonucleosomes (p < 0.01). Finally, cells in neurospheres were extracted and immunoblot analysis was performed to detect activated caspase-3. Activated caspase-3 was increased in neurospheres formed by cells in which *PLAUR* was silenced. Taken together, these results demonstrate that uPAR promotes survival of glioblastoma cells within neurospheres.

## Discussion

In the absence of pathological processes, *PLAUR* gene expression in cells in normal adult mammals is limited^54–56^. uPAR is detected mainly in tissue undergoing remodeling and in activated or migrating cells responding to injury, including inflammatory cells and activated endothelium. Many resting and quiescent cells are entirely uPAR-negative. *PLAUR* is more robustly expressed in tissues during development when the need for cell migration is increased, for example in the nervous system^57^. *PLAUR* gene expression also is increased in non-malignant cells under hypoxic conditions^55,58^. Under these circumstances, the ability of uPAR to promote cell survival may be very important. However, *PLAUR* gene deletion in mice does not have obvious effects on fertility or development^59^.

The ability of uPAR to facilitate cell survival, cell migration, and tissue invasion are exploited by cancer cells in diverse forms of malignancy^7,54,60^. By mining large TCGA datasets, we demonstrated that high levels of *PLAUR* mRNA expression are associated with a worsened prognosis in glioblastoma. Our analysis was performed with three distinct patient populations to assure that the correlation between high *PLAUR* mRNA expression and decreased patient survival did not reflect uPAR functioning as an indirect biomarker of glioma grade or a specific subtype of glioblastoma. The correlation of *PLAUR* mRNA expression with decreased patient survival in glioblastoma is particularly interesting because variability in survival in patients with this tumor is small, especially when GCIMP tumors are excluded^29,42,43^. Our analyses of patient survival did not account for patient gender, age, or treatment protocols. These parameters could feasibly have affected the results.

By IHC, we demonstrated that in many if not most human glioblastomas, uPAR protein is expressed at high levels by a small sub-population of the cells in the tumor. This finding distinguishes the pattern of uPAR expression in intact human tumors from that observed when glioblastomas are xenografted and maintained in SCID mice^33^. In the latter case, uPAR expression was homogeneously present or completely absent. Based on our IHC results, we propose that the effects of *PLAUR* mRNA expression on patient survival may reflect the activity of the small sub-population of uPAR-immunopositive cells in the tumor. High levels of *PLAUR* mRNA in a glioblastoma tissue sample may be observed if the density of uPAR-immunopositive cells is high. Alternatively, a high overall *PLAUR* mRNA level may be observed if the level of *PLAUR* mRNA is increased in the sub-population of uPAR-immunopositive cells.

Additional dataset mining and new experiments performed using neurosphere cell culture model systems provided novel insights into transitions that may occur in glioblastoma cells when these cells express high levels of uPAR. First, our results demonstrated that uPAR serves not only as a biomarker of the mesenchymal subtype of glioblastoma but also appears capable of regulating expression of other genes that, in glioblastoma cells, serve as biomarkers of the mesenchymal or proneural subtype. Mesenchymal stem cells that populate neurospheres are reported to be more aggressive when examined in *in vitro* assays and in intracranial xenografts in mice^46^. The effects of regulating *PLAUR* gene expression in TS-543, GSC-23, and U87 cells suggest that the glioblastoma gene expression signature and subtype may be regulated at the cellular level. It is important to note that *PLAUR* gene expression in cancers may be increased in hypoxia^12^. Indirectly, through its effects on *PLAUR* gene expression, hypoxia may favor transitioning of glioblastoma cells towards the mesenchymal subtype. How uPA affects uPAR-signaling and gene expression in glioblastoma cells to regulate subtype remains to be determined.

In many tumors, cancer cells with mesenchymal gene expression profiles demonstrate cancer stem cell-like properties^3,4,29^. In breast cancer cells, uPAR-activated cell-signaling induces EMT and stem cell-like properties^12,13,19^. Glioblastoma cells with stem cell-like properties have been implicated in neurosphere formation *in vitro* and may be selected for in neurosphere culture^35–37,44,45,49^. It is thus intriguing that uPAR protein expression was substantially increased in U87 cells when these cells were cultured in neurospheres as opposed to monolayers. Similarly, uPAR protein expression was substantially increased when U87vIII cells were cultured in neurospheres^53^. These results may be interpreted to indicate that U87 and U87vIII cells that are capable of seeding neurospheres are uPAR-enriched. *PLAUR* gene-silencing in U87 and U87vIII cells decreased the frequency of neurosphere formation in limiting dilution assays, reflecting a decrease in the percentage of cells capable of initiating neurosphere formation, a decrease in cell proliferation within neurospheres, and/or a decrease in cell survival in neurospheres.

We demonstrated that uPAR promotes glioblastoma cell survival in neurospheres. Although a role for uPAR in cancer cell survival is previously described together with the responsible cell-signaling pathways^16–18,32–34^, the results presented here are novel for a number of reasons. First, uPAR played a key role in promoting cell survival in U87vIII cells despite the presence of EGFRvIII and constitutively activated EGFR signaling. In monolayer culture, uPAR functions in glioblastoma cell survival mainly after EGFR signaling is neutralized^32,34^. The increase in uPAR expression and its demonstrated role in preventing cell death in neurospheres may reflect the challenging microenvironment encountered by cells in neurospheres in culture medium that is serum-free. Furthermore, compared with cells in monolayer culture, cells in neurospheres do not benefit from matrix-binding integrins that activate pro-survival cell-signaling pathways and prevent a form of apoptosis called anoikis^61^. That uPAR may contribute resistance to apoptosis in cancer cells with stem cell-like properties is an exciting hypothesis.

Finally, the effects of *PLAUR* mRNA expression on patient survival in a tumor type in which only a small sub-population of the cells express uPAR protein raises the question of whether one cancer cell in a malignancy can make adjacent cells more aggressive. In glioblastoma, there is evidence for this phenomenon. Factors that may transfer from one glioblastoma cell to another and thereby increase the aggressiveness of the recipient cell include Interleukin-6 and Leukemia Inhibitory Factor^62,63^. uPAR is released in soluble form from glioblastoma cells that express this receptor. The soluble form of uPAR is biologically active in glioblastoma and may increase the aggressiveness of other cancer cells in the tumor microenvironment^64^.

## Materials and Methods

### Mining The Cancer Genome Atlas (TCGA)

Microarray-based gene expression data and clinical annotation for Grade II, III and IV gliomas were downloaded from the publication page in TCGA^40^. RNA-Seq-based gene expression data (RNAseq V2 RSEM) and annotation for glioblastoma patients were downloaded using cBioPortal for Cancer Genomics^27^. The cases in each dataset were stratified into cohorts based on the level of expression of *PLAUR* mRNA as described. Patient survival in the various cohorts was compared. Kaplan-Meier survival curves were generated and analyzed using the Log-rank test to determine statistical significance.

### Silhouette plots

Silhouette plots were constructed using RStudio. RNA-Seq data for 151 glioblastoma cases were mined^27^. Genes previously shown to be expressed selectively by the classical (n = 162), mesenchymal (n = 216), neural (n = 129), and proneural (n = 178) subtypes of glioblastoma^28^ were examined. Whether mRNA expression of each gene correlated positively or negatively with *PLAUR* mRNA expression in the 151 case dataset was calculated. Individual genes were plotted in sequence from the lowest to highest Pearson r-value to generate a Silhouette plot consisting of 685 separate bars.

### Mining transcriptome data for glioblastoma cells propagated in *neurosphere* culture

Microarray-based gene expression data from NCBI GEO accession, GSE67089, were queried using the NCBI GEO2R profile graph tool to obtain sample gene expression values. Genes previously identified as biomarkers of the proneural and mesenchymal subtype of glioblastoma cell in neurospheres^46^ were analyzed in addition to *PLAUR* and *PLAU*. False discovery rate p-values were determined for differentially expressed genes.

### Antibodies and reagents

uPAR-specific monoclonal antibody 807 (R&D Systems) was used for immunoblotting studies. uPAR-specific antibody for IHC was from Cell Signaling Technologies. Antibodies that recognize cleaved (activated) caspase-3 and β-actin also were from Cell Signaling Technologies. GAPDH-specific antibody was from Thermo-Fisher. EGFR-specific antibody was from EMD-Millipore. B27 supplement was from Thermo-Fisher. Basic FGF and EGF were from Sigma-Aldrich.

### Cell lines and cell culture systems

U87 and HEK-293T cells were obtained from the American Type Culture Collection. EGFRvIII-expressing U87 cells are previously described^52^. All experiments were conducted within ten passages of the original stock. Cells were pre-screened for mycoplasma contamination using a mycoplasma contamination kit (Lonza). GSC-23, TS-576, GBM-6 and TS-543 glioblastoma cells, which had been passaged exclusively in neurosphere culture, were kindly provided by Dr. Frank Furnari (UCSD Ludwig Cancer Center). 627 and Mes glioblastoma cells, which had been passaged exclusively in neurosphere culture, were kindly provided by Dr. Angelo Vescovi (University of Milan Bicocca, Milan, Italy). Neurospheres were generated from GSC-23, TS-576, GBM-6, TS-543, 627, and Mes cells using the NeuroCult™ NS-A Proliferation Kit (Stem Cell Technology). Neurospheres were generated with U87 and U87vIII cells by introducing these cells into serum-free medium containing DMEM-F12 with 1 × B27 supplement, basic FGF (20 ng/mL), and EGF (20 ng/mL). Cell lines, derived from human tissue, were studied without patient identifiers. This work was reviewed and approved by the UCSD Institutional Review Board as part of the Human Research Protection Program.

### uPAR over-expression and repression/gene-silencing

A construct in which the full-length cDNA encoding human uPAR is cloned into pcDNA 3.1 (pcDNA-uPAR) and empty pcDNA 3.1 vector are previously described^65^. These plasmids were transfected into TS-543 cells in intact neurospheres (0.5 µg/10^6^ cells) using Amaxa Nucleofector (Lonza), Nucleofector Solution V, and the T-020 program. After 24 h, neurospheres were harvested for analysis.

Stable shRNA-mediated knockdown of *PLAUR* in U87 and U87vIII cells was performed using the target uPAR sequence: 5′-AGCCGTTACCTCGAATGCA-3′, which corresponds to nucleotides 397–419 within exon 4. The shRNA was cloned into the transfer vector, pLKO.1. HEK-293T cells were transfected with the transfer vector and with two separate lentivirus packaging vectors using the Addgene third generation lentivirus production system. Control viral particles were constructed with empty transfer vector. Viral particles were collected from conditioned medium. U87 and U87vIII cells were transduced by incubation with virus for 24 h. Cells were selected and maintained continuously in 1 µg/mL puromycin. Stable *PLAUR* knockdown was confirmed by RT-qPCR and immunoblot analysis 3 weeks after transduction.

*PLAUR* was silenced in U87vIII cells in neurospheres by siRNA transfection. *PLAUR*-targeting siRNA1 is previously described^9^ and targets the same sequence as the uPAR shRNA. *PLAUR*-targeting siRNA2 was a DsiRNA (Integrated DNA Technologies), which targets a sequence within exon 3: 5′-GCTATCGGACTGGCTTG-3′. Control cells were transfected with NTC On-target Plus SMARTpool siRNA (Thermo). Intact neurospheres were transfected with 50 nM siRNA using DharmaFECT 1 transfection reagent (General Electric), according to the manufacturer’s instructions.

*PLAUR* knockdown in GSC-23 cells in neurospheres was achieved using the CRISPR/Cas-9 system. A *PLAUR*-targeting gRNA sequence was designed to minimize off-target effects using the gRNA design tool from DNA 2.0. An oligonucleotide cassette was synthesized and cloned into the Cas-9 vector, pD1321-APuro (DNA2.0), using SAP-1 restriction sites. The oligonucleotide cassette sequence was 5′-TACACGTACTAGTCGC-TGAAGCTCTTCACCGGACCACGATCGTGCGCTTGTGTTAGAAGAGCCGTCAATCGAGTTCGTACCT-3′. The *PLAUR*-targeting gRNA is underlined. The Cas-9-*PLAUR* vector or a pCas-9 scrambled control vector (Origene) was transfected into GSC-23 cells in intact neurospheres (0.5 µg/10^6^ cells) using Amaxa Nucleofector (Lonza), Nucleofector Solution V, and the T-020 program. After 24 h, neurospheres were collected for analysis.

### RT-qPCR3

Total RNA was isolated from cells in neurospheres using the NucleoSpin RNA II kit (Macherey-Nagel). cDNA was synthesized using the iScript cDNA synthesis kit (Bio-Rad). PCR was performed using TaqMan Fast Universal PCR Mastermix (Applied Biosystems) on a System 7300 Applied Biosystems instrument with the following TaqMan primers and probes from Life Technologies: human *PLAUR* (00959822), human *PLAU* (01547054), human *CD44* (01075864), human *BCL2A1* (00187845), human *LYN* (01015816), human *CD133* (01009257), human *SOX2* (01053049), human *NOTCH1* (01062014), and human *HPRT1* (02800695). mRNA expression was standardized relative to *HPRT1* mRNA using the 2^−ΔΔCt^ method.

### Immunoblot analysis

Extracts of cells were prepared in RIPA buffer (PBS, 1% Triton X-100, 0.5% sodium deoxycholate, and 0.1% SDS) supplemented with EDTA-free protease inhibitor mixture (Thermo Scientific). Protein in cell extracts was determined by bicinchoninic acid assay. Equal amounts of cell extract were loaded in each lane (35 μg) for SDS-PAGE. Proteins were electrotransferred to 0.45-μm PVDF membranes and incubated with primary antibodies followed by horseradish peroxidase-conjugated secondary antibodies. Signal was developed using SuperSignal West Pico (Pierce) or Femto substrate (Thermo-Fisher Scientific). For immunoblots in which relative band intensity is reported, at least three separate blots were subjected to densitometry using Image J.

### WST-1 cell viability assay

U87vIII cells were plated at a density of 10,000 cells per well in 12 well plates in medium supplemented with 10% FBS. At the indicated times, WST-1 reagent was incubated with the cells for 1 h. Formazan dye formation was quantitated by measuring absorbance at 450 nm using a Spectramax M2 scanning multiwall spectrophotometer.

### Neurosphere formation and growth assays

In limiting dilution neurosphere formation assays, cells were plated at a density of 1–100 cells per well in 96-well plates in neurosphere medium. Neurospheres were allowed to form undisturbed for 14 days, at which time, the number of neurospheres that achieved a minimum diameter of 60 µm was determined by phase contrast microscopy. Experiments were performed in triplicate. In each experiment, plating densities were studied in quadruplicate. Results were fit by linear regression using GraphPad Prism (r^2^ values always exceed 0.9).

For neurosphere volume analysis, U87vIII cells were suspended in neurosphere medium at a density of 10,000 cells/cm^2^. Neurospheres were allowed to develop for 6 days. Neurosphere images were acquired at 100× magnification. The mean radius of each neurosphere was determined using Image-J software (NIH). The volume of the sphere, which reports cellular content assuming close packing of the cells, was then calculated according to the equation: V = (4/3)πr^3^. All of the neurospheres in three wells were measured in three separate experiments.

### Analysis of apoptosis

Neurospheres were allowed to form for 6 days. The cells were then harvested and apoptosis was assessed using the Roche Cell Death Detection ELISA-plus kit. Protein extracts were subjected to immunoblot analysis to detect activated caspase-3. In separate experiments, we analyzed apoptosis in intact neurospheres by TUNEL using the APO-BrdU TUNEL Assay Kit (Molecular Probes) according to the manufacturer’s protocol. Briefly, intact neurospheres were fixed in 4% paraformaldehyde and subjected to the TUNEL reaction using Br-UTP to label fragmented DNA associated with apoptosis. Apoptotic cells were detected using an antibody raised against BrdU. Because the cells were fixed before being exposed to Br-UTP, the BrdU antibody only identifies apoptotic cells and not proliferating cells in S-phase. To assess the fraction of cells undergoing apoptosis in intact neurospheres, 25 randomly selected neurospheres were selected for each condition. The ratio of TUNEL-positive cells to the total number of DAPI-stained nuclei was determined.

### IHC

Tissue blocks were obtained without patient identifiers after the diagnosis of glioblastoma was confirmed by a neuropathologist, according to an IRB-approved protocol. Four micron-thick tissue sections were immunostained with uPAR-specific antibody. Immunostaining was performed using a Ventana Discovery Ultra (Ventana Medical Systems, Tucson, AZ, USA). Antigen retrieval was performed using CC1 for 40 minutes at 95 °C. IHC staining was followed by hematoxylin counterstaining. Slides were rinsed, dehydrated though alcohol and xylene, and cover-slipped.

### Statistics

Statistical analysis was performed using GraphPad Prism 5. All results reflect at least three independent experiments.

## Electronic supplementary material

Supplemental Data

## References

[1] Thiery JP, Acloque H, Huang RY, Nieto MA (2009). Epithelial-mesenchymal transitions in development and disease. Cell..

[2] Thiery JP, Sleeman JP (2006). Complex networks orchestrate epithelial-mesenchymal transitions. Nat Rev Mol Cell Biol..

[3] Mani SA (2008). The epithelial-mesenchymal transition generates cells with properties of stem cells. Cell..

[4] Singh A, Settleman J (2010). EMT, cancer stem cells and drug resistance, an emerging axis of evil in the war on cancer. Oncogene..

[5] Yu M (2013). Circulating breast tumor cells exhibit dynamic changes in epithelial and mesenchymal composition. Science..

[6] Blasi F, Vassalli JD, Dano K (1987). Urokinase-type plasminogen activator, proenzyme, receptor, and inhibitors. J Cell Biol..

[7] Andreasen PA, Kjoller L, Christensen L, Duffy MJ (1997). The urokinase-type plasminogen activator system in cancer metastasis, a review. Int J Cancer..

[8] Ploug M, Behrendt N, Lober D, Dano K (1991). Protein structure and membrane anchorage of the cellular receptor for urokinase-type plasminogen activator. Semin Thromb Hemost..

[9] Blasi F, Carmeliet P (2002). uPAR, a versatile signalling orchestrator. Nat Rev Mol Cell Biol..

[10] Jo M (2005). Dynamic assembly of the urokinase-type plasminogen activator signaling receptor complex determines the mitogenic activity of urokinase-type plasminogen activator. J Biol Chem..

[11] Smith HW, Marshall CJ (2010). Regulation of cell signalling by uPAR. Nat Rev Mol Cell Biol..

[12] Lester RD, Jo M, Montel V, Takimoto S, Gonias SL (2007). uPAR induces epithelial-mesenchymal transition in hypoxic breast cancer cells. J Cell Biol..

[13] Jo M (2009). Reversibility of epithelial-mesenchymal transition (EMT) induced in breast cancer cells by activation of urokinase receptor-dependent cell signaling. J Biol Chem..

[14] Nguyen DH, Hussaini IM, Gonias SL (1998). Binding of urokinase-type plasminogen activator to its receptor in MCF-7 cells activates extracellular signal-regulated kinase 1 and 2 which is required for increased cellular motility. J Biol Chem..

[15] Webb DJ, Nguyen DH, Gonias SL (2000). Extracellular signal-regulated kinase functions in the urokinase receptor-dependent pathway by which neutralization of low density lipoprotein receptor-related protein promotes fibrosarcoma cell migration and matrigel invasion. J Cell Sci..

[16] Alfano D (2005). The urokinase plasminogen activator and its receptor, role in cell growth and apoptosis. Thromb Haemost..

[17] Ma Z, Webb DJ, Jo M, Gonias SL (2001). Endogenously produced urokinase-type plasminogen activator is a major determinant of the basal level of activated ERK/MAP kinase and prevents apoptosis in MDA-MB-231 breast cancer cells. J Cell Sci..

[18] Alfano D, Iaccarino I, Stoppelli MP (2006). Urokinase signaling through its receptor protects against anoikis by increasing BCL-xL expression levels. J Biol Chem..

[19] Jo M (2010). Cell signaling by urokinase-type plasminogen activator receptor induces stem cell-like properties in breast cancer cells. Cancer Res..

[20] Jo M, Takimoto S, Montel V, Gonias SL (2009). The urokinase receptor promotes cancer metastasis independently of urokinase-type plasminogen activator in mice. Am J Pathol..

[21] Pirazzoli V, Ferraris GM, Sidenius N (2013). Direct evidence of the importance of vitronectin and its interaction with the urokinase receptor in tumor growth. Blood..

[22] Eastman BM, Jo M, Webb DL, Takimoto S, Gonias SL (2012). A transformation in the mechanism by which the urokinase receptor signals provides a selection advantage for estrogen receptor-expressing breast cancer cells in the absence of estrogen. Cell Signal..

[23] Omuro A, DeAngelis LM (2013). Glioblastoma and other malignant gliomas, a clinical review. JAMA..

[24] Furnari FB (2007). Malignant astrocytic glioma, genetics, biology, and paths to treatment. Genes Dev..

[25] The Cancer Genome Atlas Research Network. Comprehensive genomic characterization defines human glioblastoma genes and core pathways. *Nature*. **455**, 1061–1068 (2008). 10.1038/nature07385 PMC2671642 18772890

[26] Dunn GP (2012). Emerging insights into the molecular and cellular basis of glioblastoma. Genes Dev..

[27] Brennan CW (2013). The somatic genomic landscape of glioblastoma. Cell..

[28] Verhaak RG (2010). Integrated genomic analysis identifies clinically relevant subtypes of glioblastoma characterized by abnormalities in PDGFRA, IDH1, EGFR, and NF1. Cancer Cell..

[29] Phillips HS (2006). Molecular subclasses of high-grade glioma predict prognosis, delineate a pattern of disease progression, and resemble stages in neurogenesis. Cancer Cell..

[30] Sullivan JP (2014). Brain tumor cells in circulation are enriched for mesenchymal gene expression. Cancer Discov..

[31] Patel AP (2014). Single-cell RNA-seq highlights intratumoral heterogeneity in primary glioblastoma. Science..

[32] Hu J (2011). Crosstalk between the urokinase-type plasminogen activator receptor and EGF receptor variant III supports survival and growth of glioblastoma cells. Proc Natl Acad Sci USA.

[33] Hu J (2014). Neutralizing the EGF receptor in glioblastoma cells stimulates cell migration by activating uPAR-initiated cell signaling. Oncogene..

[34] Wykosky J (2015). A urokinase receptor-Bim signaling axis emerges during EGFR inhibitor resistance in mutant EGFR glioblastoma. Cancer Res..

[35] Gunther HS (2008). Glioblastoma-derived stem cell-enriched cultures form distinct subgroups according to molecular and phenotypic criteria. Oncogene..

[36] Laks DR (2009). Neurosphere formation is an independent predictor of clinical outcome in malignant glioma. Stem Cells..

[37] Chen R (2010). A hierarchy of self-renewing tumor-initiating cell types in glioblastoma. Cancer Cell..

[38] Yamamoto M (1994). Expression and localization of urokinase-type plasminogen activator in human astrocytomas *in vivo*. Cancer Res..

[39] Salajegheh M, Rudnicki A, Smith TW (2005). Expression of urokinase-type plasminogen activator receptor (uPAR) in primary central nervous system neoplasms. Appl Immunohistochem Mol Morphol..

[40] Ceccarelli M (2016). Molecular Profiling Reveals Biologically Discrete Subsets and Pathways of Progression in Diffuse Glioma. Cell..

[41] Wang Z, Gerstein M, Snyder M (2009). RNA-Seq, a revolutionary tool for transcriptomics. Nat Rev Genet..

[42] Noushmehr H (2010). Identification of a CpG island methylator phenotype that defines a distinct subgroup of glioma. Cancer Cell..

[43] Turcan S (2012). IDH1 mutation is sufficient to establish the glioma hypermethylator phenotype. Nature..

[44] Galli R (2004). Isolation and characterization of tumorigenic, stem-like neural precursors from human glioblastoma. Cancer Res..

[45] Weiswald LB, Bellet D, Dangles-Marie V (2015). Spherical cancer models in tumor biology. Neoplasia.

[46] Mao P (2013). Mesenchymal glioma stem cells are maintained by activated glycolytic metabolism involving aldehyde dehydrogenase 1A3. Proc Natl Acad Sci USA.

[47] Brown DV (2015). Coexpression analysis of CD133 and CD44 identifies proneural and mesenchymal subtypes of glioblastoma multiforme. Oncotarget..

[48] Lottaz C (2010). Transcriptional profiles of CD133+ and CD133− glioblastoma-derived cancer stem cell lines suggest different cells of origin. Cancer Res..

[49] Yu SC (2008). Isolation and characterization of cancer stem cells from a human glioblastoma cell line U87. Cancer Lett..

[50] Heimberger AB (2005). Prognostic effect of epidermal growth factor receptor and EGFRvIII in glioblastoma multiforme patients. Clin Cancer Res..

[51] Sugawa N, Ekstrand AJ, James CD, Collins VP (1990). Identical splicing of aberrant epidermal growth factor receptor transcripts from amplified rearranged genes in human glioblastomas. Proc Natl Acad Sci USA.

[52] Nishikawa R (1994). A mutant epidermal growth factor receptor common in human glioma confers enhanced tumorigenicity. Proc Natl Acad Sci USA.

[53] Gilder AS (2016). Pertussis Toxin Is a Robust and Selective Inhibitor of High Grade Glioma Cell Migration and Invasion. PLoS One..

[54] Mazar AP (2008). Urokinase plasminogen activator receptor choreographs multiple ligand interactions, implications for tumor progression and therapy. Clin Cancer Res..

[55] Gonias SL, Hu J (2015). Urokinase receptor and resistance to targeted anticancer agents. Front Pharmacol.

[56] Solberg H, Ploug M, Hoyer-Hansen G, Nielsen BS, Lund LR (2001). The murine receptor for urokinase-type plasminogen activator is primarily expressed in tissues actively undergoing remodeling. J Histochem Cytochem..

[57] Merino P, Diaz A, Yepes M (2017). Urokinase-type plasminogen activator (uPA) and its receptor (uPAR) promote neurorepair in the ischemic brain. Receptors Clin Investig..

[58] Graham CH, Fitzpatrick TE, McCrae KR (1998). Hypoxia stimulates urokinase receptor expression through a heme protein-dependent pathway. Blood..

[59] Bugge TH (1995). The receptor for urokinase-type plasminogen activator is not essential for mouse development or fertility. J Biol Chem..

[60] Dano K (2005). Plasminogen activation and cancer. Thromb Haemost..

[61] Frisch SM, Ruoslahti E (1997). Integrins and anoikis. Curr Opin Cell Biol..

[62] Inda MM (2010). Tumor heterogeneity is an active process maintained by a mutant EGFR-induced cytokine circuit in glioblastoma. Genes Dev..

[63] Zanca, C. *et al*. Glioblastoma cellular cross-talk converges on NF-kappaB to attenuate EGFR inhibitor sensitivity. *Genes Dev*. 10.1101/gad.300079.117 (2017). 10.1101/gad.300079.117 PMC5558924 28724615

[64] Gilder AS (2015). Soluble urokinase receptor is released selectively by glioblastoma cells that express epidermal growth factor receptor variant III and promotes tumor cell migration and invasion. J Biol Chem..

[65] Jo M, Thomas KS, O’Donnell DM, Gonias SL (2003). Epidermal growth factor receptor- dependent and -independent cell-signaling pathways originating from the urokinase receptor. J Biol Chem..

