# Supplementary material for: The Urokinase Receptor Induces a Mesenchymal Gene Expression Signature in Glioblastoma Cells and Promotes Tumor Cell Survival in Neurospheres
Source: Sci Rep. 2018 Feb 14;8:2982. doi: 10.1038/s41598-018-21358-1 (PMC5813209; doi:10.1038/s41598-018-21358-1)
Supplement: Supplementary file 1 — Supplemental Data [file 41598_2018_21358_MOESM1_ESM.pdf]

The Urokinase Receptor Induces a Mesenchymal Gene Expression Signature in Glioblastoma Cells and Promotes Tumor Cell Survival in Neurospheres

Andrew S. Gilder, Letizia Natali, Danielle M. Van Dyk, Cristina Zalfa, Michael A. Banki, Donald P. Pizzo, Huawei Wang, Richard L. Klemke, Elisabetta Mantuano, and Steven L. Gonias

Supplemental Figures 1 and 2

Supplemental Fig. 1

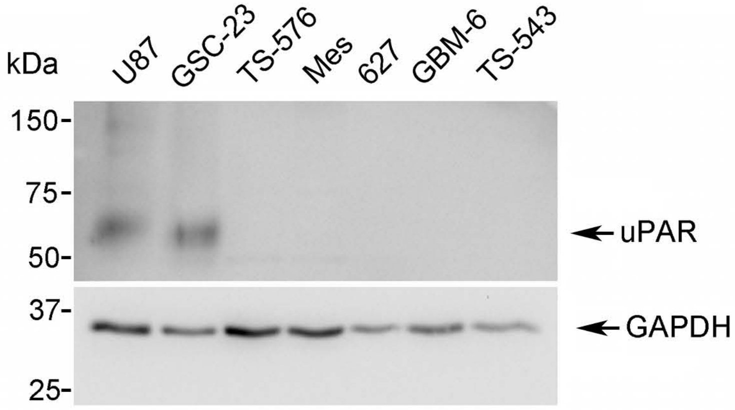

Supplemental Figure 1. Original immunoblot shown in Fig. 5C.

Supplemental Fig. 2

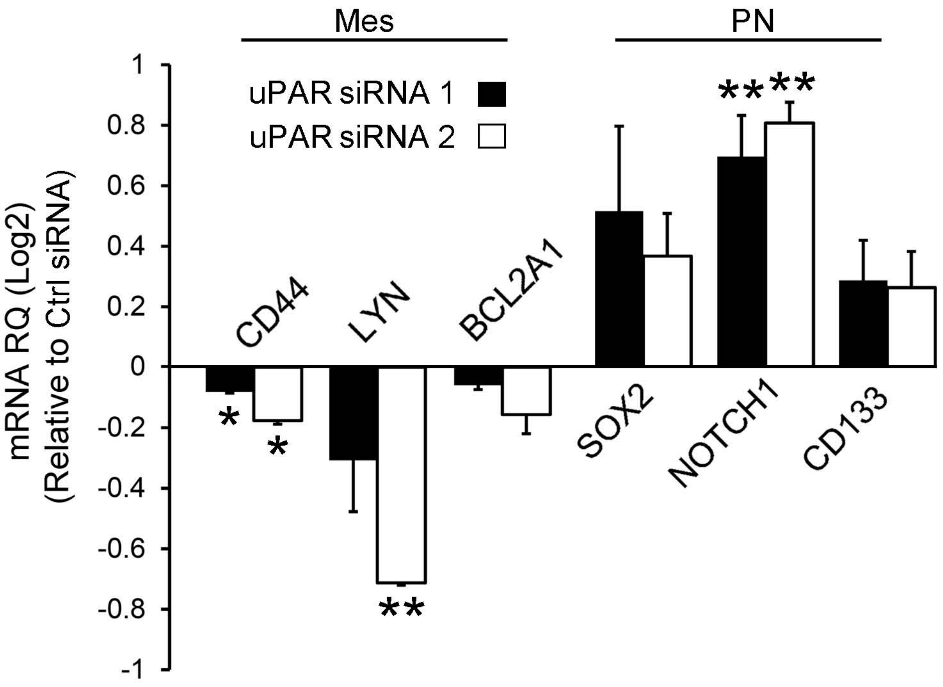

Supplemental Figure 2. RT-qPCR was performed to determine relative mRNA expression for *CD44*, *LYN*, *BCL2A1*, *SOX2*, *NOTCH1*, and *CD133* in U87vIII neurospheres transiently expressing *PLAUR*-targeting siRNA (siRNA1, siRNA2) versus NTC siRNA (Ctrl siRNA) (mean ± S.E., n=3; student's t-test **p<0.01; p<0.05).
